# Cancer stem cells, tumor dormancy, and metastasis

**DOI:** 10.3389/fendo.2012.00125

**Published:** 2012-10-23

**Authors:** Purvi Patel, Emily I. Chen

**Affiliations:** Department of Pharmacological Sciences, Stony Brook UniversityStony Brook, NY, USA

**Keywords:** cancer stem cells, metastasis, tumor dormancy, stem-like subpopulations, disseminated CSCs, EMT–MET cooperativity

## Abstract

Tumor cells can persist undetectably for an extended period of time in primary tumors and in disseminated cancer cells. Very little is known about why and how these tumors persist for extended periods of time and then evolve to malignancy. The discovery of cancer stem cells (CSCs) in human tumors challenges our current understanding of tumor recurrence, drug resistance, and metastasis, and opens up new research directions on how cancer cells are capable of switching from dormancy to malignancy. Although overlapping molecules and pathways have been reported to regulate the stem-like phenotype of CSCs and metastasis, accumulated evidence has suggested additional clonal diversity within the stem-like cancer cell subpopulation. This review will describe the current hypothesis linking CSCs and metastasis and summarize mechanisms important for metastatic CSCs to re-initiate tumors in the secondary sites. A better understanding of CSCs’ contribution to clinical tumor dormancy and metastasis will provide new therapeutic revenues to eradicate metastatic tumors and significantly reduce the mortality of cancer patients.

## INTRODUCTION

Solid tumors account for the major cancer burden, and epithelial cancers arising in breast, lung, colon, prostate, and ovary comprise approximately 80% of all cancers (Visvader and Lindeman, 2008). However, over 90% of mortality in cancer patients is attributed to the subsequent spread of cancer cells to distant tissues (Weigelt et al., 2005; Jemal et al., 2006; Steeg, 2006). In patients, the threat of tumor can return after chemotherapy and radiation remains terrifying and painfully real. In breast cancer, for example, metastasis can occur after decades of apparent disease-free period prior to the development of distant metastasis (Meltzer, 1990; Uhr et al., 1997). This phenomenon is referred to as clinical tumor dormancy and is frequently observed in cancer patients. Both clinical observations and experimental models have revealed that cancer patients may have hundreds to thousands of disseminated cancer cells detectable in circulation but only a small portion of the disseminated cancer cells progress to form clinically overt metastases (Tarin et al., 1984; Weiss, 1990, 1992).

Metastasis is a multi-step process. It is well recognized that cancer cells capable of metastasizing acquire epithelial–mesenchymal transition (EMT)-like phenotype allowing them to disseminate from the primary tumor and intravasate into the circulation. In circulation, these disseminated cancer cells have to survive and then eventually extravasate into foreign tissues. Finally, few of these cells will adapt in the microenvironment and form macrometastases. Experimental evidence has demonstrated that early step of metastasis (intravasate, survival, arrest, and extravasation) can be highly efficient (Cameron et al., 2000; Suzuki et al., 2006). However, only a small subset of these cells (~2%) can initiate growth as micrometastases, and an even smaller fraction of these cells (~0.02%) are able to persist and form macrometastases (Cameron et al., 2000; Chambers et al., 2000, 2001; Suzuki et al., 2006). Therefore, the later steps of metastasis appear to be the most critical steps for metastatic dormancy. In spite of the clinical importance of metastasis, the mechanisms underlying the process of dormancy and outgrowth of macrometastases remain poorly understood.

Accumulating evidence suggests that a subpopulation of cancer cells exhibit stem-like properties and is capable of tumor initiation, invasive growth, and disseminating to distant organs (Reya et al., 2001; Li et al., 2007; Liu et al., 2010; Marotta and Polyak, 2009; O’Brien et al., 2009). These cancer stem cells (CSCs) have the ability to self-renew to give rise to other tumorigenic cells, as well as undergo differentiation to give rise to the phenotypically diverse non-tumorigenic cancer cells. Several characteristics of CSCs, including the phenotypic plasticity, make them more likely to succeed in the later steps of metastasis. This review will focus on specific stem cell features of CSCs relating to cancer metastasis and implications of CSC theory on treatment strategies against metastasis.

## STEM CELL CHARACTERISTICS RELATING TO TUMOR DISSEMINATION

It is well recognized that some cancer cells are capable of undergoing an EMT-like transformation and develop a migratory and invasive phenotype to detach from the primary tumor (Kang and Massague, 2004; Micalizzi et al., 2010; Gomes et al., 2011; Nauseef and Henry, 2011; Said and Williams, 2011; Yao et al., 2011). EMT is a biologic process that allows epithelial cells to undergo multiple biochemical changes that acquire a mesenchymal cell phenotype with an enhanced migratory capacity, invasiveness, elevated resistance to apoptosis, and increased production of ECM components (Kalluri, 2009; Yilmaz and Christofori, 2009). CSCs have been hypothesized to be the disseminating subpopulation and supported by accumulating evidence that CSCs also express EMT markers, and more importantly, induction of EMT in transformed epithelial cells promotes the generation of CSCs (Yang et al., 2004; Mani et al., 2008; Floor et al., 2011; Jordan et al., 2011; Wu, 2011; Wu and Yang, 2011; Krantz et al., 2012). For example, in colon cancer, nuclear accumulation of β-catenin, the feature of Wnt signaling activation and stem cell signaling, is found at the invasive front of the primary tumor (Fodde and Brabletz, 2007). Stem-like cells isolated from normal mammary glands and breast tumors also express EMT markers (Damonte et al., 2007; Mani et al., 2008). Stem-like breast cancer cells isolated from exposing breast cancer cells to cycles of hypoxia and reoxygenation also show up-regulation of several EMT-inducing factors (Louie et al., 2010). Overexpression of EMT-inducing transcription factors such as Snail or Twist, in transformed mammary epithelial cells (HMLEM) increased tumor-initiating frequency in immune-deficient mice (Mani et al., 2008). Similar to the embryonic programming of EMT, activation of EMT signaling in CSCs is also regulated by a reciprocal feedback loop between the ZEB family of EMT inducers and miR-200 family of EMT suppressors (Hurteau et al., 2007; Burk et al., 2008; Gregory et al., 2008; Korpal et al., 2008; Park et al., 2008) as well as a second reciprocal feedback loop with EMT suppressor, miR-34, and EMT inducer, Snail (Kim et al., 2011; Siemens et al., 2011). Together, these observations lend support to the model of migrating CSCs that confer the plasticity of switching between cellular states and detaching efficiently from the primary tumor.

## STEM CELL CHARACTERISTICS RELATING TO DORMANCY AND METASTASIS

In addition to the self-renewing and EMT phenotypes, CSCs also exhibit other stem cell properties that are beneficial for them to adapt in the foreign microenvironment and eventually form clinical overt metastasis. It is apparent that several unique properties necessary for ensuring long life span of normal stem cells may contribute to protection of CSCs in the adverse microenvironment. For example, normal stem cells have increased capacity for DNA repair and express higher levels of anti-apoptotic proteins than differentiated cells (Cairns, 2002; Potten et al., 2002; Wang et al., 2003; Park and Gerson, 2005; Shinin et al., 2006). After disseminated cancer cells arrive at the foreign microenvironment, the suppressive target organ can form a barrier to halt the progression of metastasis and induce dormancy. The enhanced anti-apoptotic and DNA repair capability of CSCs could increase the survival of CSCs for a long period of time under metabolic and/or other environmental stress (e.g., hypoxia) in the target organ and allow them to find adaptive solutions.

Evidence supporting this model comes from studies showing CSCs have increased drug resistance capacity. For example, it has been shown that stem-like subpopulation of cancer cells express high levels of ATP-binding cassette (ABC) transporters that can actively efflux drugs and shield them from the adverse effects of chemotherapeutic insult (Pardal et al., 2003; Lou and Dean, 2007; Dean, 2009; Donnenberg et al., 2009; Ding et al., 2010; Moitra et al., 2011). In addition to an increased drug efflux capacity, CSCs also exhibit intrinsic resistance to apoptosis. For instance, autocrine production of cytokines such as IL-4 has been shown to increased anti-apoptotic proteins and induces resistance to therapy-induced cytotoxicity in different cancer types (Conticello et al., 2004). For patients with colon cancer, IL-4 antagonist was shown to strongly enhance the anti-tumor efficacy of conventional chemotherapeutic drugs through selective sensitization of colon CSCs that are CD133^+^ (Todaro et al., 2007). There is also growing evidence that CSCs are inherently resistant to radiation (Koepke, 2006; Rich, 2007; Debeb et al., 2009; Pajonk et al., 2010; Croker and Allan, 2012; D’Andrea, 2012). For example, the effectiveness of radiotherapy is mediated by the induction of reactive oxygen species (ROS) in cancer cells. However, it has been found that both human and mouse mammary CSCs contain lower ROS levels than more differentiated tumor cells and accumulate less DNA damage upon radiation (Diehn et al., 2009). Lower ROS levels in CSCs appear to result from increased expression of free radical scavenging systems (Diehn et al., 2009; Wang et al., 2010; Kobayashi and Suda, 2012; Shi et al., 2012). Together, the experimental evidence is consistent with clinical observations that drug resistance is seemingly higher in metastatic disease. More importantly, the inherent feature of drug resistance in CSCs could activate stress responses to protect them from growth-suppressing conditions in the target organ microenvironment and allow them to persist in foreign tissues for a long period of time.

## STEM CELL CHARACTERISTICS RELATING TO THE FORMATION OF CLINICALLY OVERT METASTASIS

It has been observed that a high proportion of distant metastases are differentiated and in some cases metastases can show a greater degree of cellular differentiation than the primary tumors. For example, increased E-cadherin expression in metastases compared to the primary tumors has been reported in human patient specimens (Oka et al., 1993; Kowalski et al., 2003; Chao et al., 2010). Furthermore, the importance of epithelial phenotype in the formation of secondary tumors has been demonstrated in different metastasis models, including bladder cancer (Chaffer et al., 2005, 2006, 2007), prostate cancer (Oltean et al., 2006; Yates et al., 2007), colorectal cancer (Vincan et al., 2007a,b), and breast cancer (Tsuji et al., 2008, 2009; Chao et al., 2010). Hence, both clinical and experiment evidence points to the necessity of disseminated cancer cells undergoing a mesenchymal to epithelial reverting transition (MET) in the secondary microenvironment to form macrometastases. Consequently, it has been proposed that metastatic cancer cells possess the phenotypic plasticity and acquired EMT-like phenotype for disseminating from the primary tumor and subsequently, a second transition from the EMT-like to MET-like state occurs to facilitate the formation of metastatic tumors at target organs (Brabletz, 2012). However, it is unclear whether EMT/stem-like cancer cells (CSCs) cooperate with non-EMT cancer cells for successful colonization at target organs or CSCs maintain a fused cell phenotype with incomplete or mixed epithelial characteristics that can be used to initiate the subsequent adaptation in the foreign microenvironment. For example, Tsuji et al. (2008) showed that cancer cells with an induced EMT state increase invasiveness locally in the primary tumor but fail to promote distant colonization when introduced in circulation. Also, co-culture of hepatocytes and EMT-like prostate cancer cells resulted in up-regulation of E-cadherin in these cells (Yates et al., 2007). We also found that brain metastatic breast cancer cells utilize oxidative metabolism that is similar to the normal epithelial cells instead of the glycolytic metabolism utilized by rapidly proliferating EMT-like breast cancer cells (Chen et al., 2007).

To complicate matters further, the metastatic niche can also supplies extrinsic factors that can further influence the proliferation and cellular states of metastatic cancer cells. Proliferation of disseminated CSCs in the targeted organs can be regulated by growth-suppressing and growth-stimulating factors secreted by or into the microenvironment. For example, a single dormant cancer cell or a dormant micrometastasis can turn into clinically detectable metastasis through an increased secretion of angiogenic factors in the metastatic niche to promote the recruitment and formation of new blood vessels (angiogenesis; Takahashi and Mai, 2005; Gao et al., 2008; Raza et al., 2010; Saharinen et al., 2011; Garcia and Kandel, 2012). It has been reported that CSCs promote tumor angiogenesis by actively secreting angiogenic factors such as vascular endothelial growth factor (VEGF; Bao et al., 2006; Seton-Rogers, 2011). In addition to secreted factors, cell–cell interactions in the microenvironment can also mediate protection of disseminated CSCs in the metastatic niche. For instance, it has been shown that mesenchymal stem cells (MSCs) induce the production of regulatory T cells (T_regs_) when co-cultured with breast cancer cells allowing for immune subversion of breast cancer cells in the bone marrow (Patel et al., 2010). Also, it has been shown that bone marrow-derived MSCs could promote the expansion of CSC population in the co-culture system and accelerate tumor growth in the xenograft mouse model (Liu et al., 2011). Therefore, interactions between disseminated CSCs and tumor-associated MSCs may play a role in preventing the elimination of cancer cells by normal anti-tumor immune responses in the metastatic microenvironment.

## THERAPEUTIC CONSEQUENCES AND PERSPECTIVES OF MIGRATING CANCER STEM CELLS

The presence of a distinct population with stem cell characteristics among disseminated and circulating cancer cells has an important clinical relevance in metastasis formation and recurrence, but more importantly for their role in resistance to conventional anti-cancer therapy. The stem-like properties of CSCs have been shown to be advantageous in protecting them from environmental and genotoxic stresses. The observed phenotypic plasticity of CSCs and their inherent anti-apoptotic ability can potentially render them capable of navigating through the entire metastatic cascade. It is apparent that current therapeutic strategies fail to account for potential molecular and proliferative differences between different subpopulations of tumor cells. This may explain why the majority of therapies fail in treating patients with metastatic diseases. CSC model also has significant implications for cancer progression. It has been observed in clinics that remission generally becomes more difficult to achieve with each relapse, and the diagnosis of metastatic disease significantly reduces patient survival. This suggests that the cancer cell population becomes increasingly aggressive over time, perhaps reflecting the selectively killing of the non-tumorigenic cells and enriching for the inherently therapeutic resistant CSCs in the metastatic tumor. Indeed, accumulating experimental evidence has confirmed this suspicion and demonstrated the potential benefit of selectively targeting the stem-like subpopulation in preventing cancer progression (Gupta et al., 2009; Newman et al., 2012). However, alternative evidence opposing CSC model also exists suggesting that CSCs may not be the only cell type to sustain tumor growth and generate metastasis (Kelly et al., 2007; Yoo and Hatfield, 2008). Despite of the controversial origin or source of stem-like subpopulations, it is clear that a better definition and understanding of CSCs in clinical tumor dormancy as well as cancer relapse will have a significant impact on the mortality of patients with advanced stage of cancer.

## Conflict of Interest Statement

The authors declare that the research was conducted in the absence of any commercial or financial relationships that could be construed as a potential conflict of interest.
